# First Nations emergency care in Alberta: descriptive results of a retrospective cohort study

**DOI:** 10.1186/s12913-021-06415-2

**Published:** 2021-05-04

**Authors:** Patrick McLane, Cheryl Barnabe, Brian R. Holroyd, Amy Colquhoun, Lea Bill, Kayla M. Fitzpatrick, Katherine Rittenbach, Chyloe Healy, Bonnie Healy, Rhonda J. Rosychuk

**Affiliations:** 1 Alberta Health Services, Strategic Clinical Networks, Alberta Health Services Corporate Office, Seventh Street Plaza, 14th Floor, North Tower, 10030 – 107 Street NW, Edmonton, AB T5J 3E4 Canada; 2 Department of Emergency Medicine, University of Alberta, 790 University Terrace Building, 8303 - 112 Street, Edmonton, AB T6G 2T4 Canada; 3 Department of Medicine, Health Sciences Centre, University of Calgary, 3330 Hospital Drive NW, Calgary, AB T2N 4N1 Canada; 4 Alberta Health, Analytics and Performance Reporting, Telus Plaza North, Main Floor- 10025 Jasper Avenue NW, Edmonton, AB T5J 1S6 Canada; 5 Alberta First Nations Information Governance Centre, Suite 101, 535 8 Ave SE, Calgary, AB T2G 5S9 Canada; 6 Department of Psychiatry, Health Sciences Centre, University of Calgary, 3330 Hospital Drive NW, Calgary, AB T2N 4N1 Canada; 7 Department of Psychiatry, University of Alberta, 1E1 Walter Mackenzie Health Sciences Centre, 8440 112 St NW, Edmonton, AB T6G 2B7 Canada; 8 First Nations Health & Social Secretariat of Manitoba, Unit B103, 1075 Portage Ave, Winnipeg, MB R3G 0R8 Canada; 9 Blackfoot Confederacy, Health Services, P.O. Box 916, Standoff, AB T0L 1Y0 Canada; 10 Department of Pediatrics, University of Alberta, Edmonton Clinic Health Academy, 11405-87 Avenue, Edmonton, AB T6G 1C9 Canada

**Keywords:** Indigenous, Emergency department, Health equity, Access to care

## Abstract

**Background:**

Worse health outcomes are consistently reported for First Nations people in Canada. Social, political and economic inequities as well as inequities in health care are major contributing factors to these health disparities. Emergency care is an important health services resource for First Nations people. First Nations partners, academic researchers, and health authority staff are collaborating to examine emergency care visit characteristics for First Nations and non-First Nations people in the province of Alberta.

**Methods:**

We conducted a population-based retrospective cohort study examining all Alberta emergency care visits from April 1, 2012 to March 31, 2017 by linking administrative data. Patient demographics and emergency care visit characteristics for status First Nations persons in Alberta, and non-First Nations persons, are reported. Frequencies and percentages (%) describe patients and visits by categorical variables (e.g., Canadian Triage and Acuity Scale). Means, medians, standard deviations and interquartile ranges describe continuous variables (e.g., age).

**Results:**

The dataset contains 11,686,288 emergency care visits by 3,024,491 unique persons. First Nations people make up 4% of the provincial population and 9.4% of provincial emergency visits. The population rate of emergency visits is nearly 3 times higher for First Nations persons than non-First Nations persons. First Nations women utilize emergency care more than non-First Nations women (54.2% of First Nations visits are by women compared to 50.9% of non-First Nations visits). More First Nations visits end in leaving without completing treatment (6.7% v. 3.6%).

**Conclusions:**

Further research is needed on the impact of First Nations identity on emergency care drivers and outcomes, and on emergency care for First Nations women.

**Supplementary Information:**

The online version contains supplementary material available at 10.1186/s12913-021-06415-2.

## Background

Poor health outcomes impact Indigenous peoples internationally, as both a consequence and mechanism of colonization [1, 2]. Within Canada, the term First Nations (FN) refers to one of the three Indigenous groups recognized as holding aboriginal and treaty rights by the *Constitution Act, 1982,* alongside Inuit and Metis [3, 4]. Health disparities for FN persons in Canada [5, 6] are recognized as related to persistent social, political and economic inequities, including inequities in health care services and delivery [6–10]. Academics and government inquiries agree that colonialism and racism have led to devastating impacts on FN health [7, 11–13]. Emergency medicine has an important role in providing care to marginalized populations that have limited access to other health care services, given its longstanding commitments to providing care to all comers [14]. Non-academic and academic literature shows that Indigenous persons rely more heavily on emergency services than non-Indigenous persons [15–17].

While it is laudable that emergency medicine provides accessible care for underserved groups, disparities in emergency care for racial and ethnic minorities are well documented [18, 19]. Indigenous patients specifically have been reported to leave emergency departments more often without being seen [16, 20–22], potentially indicating dissatisfaction with care. Canadian qualitative research documents patient concerns about differential treatment based on race and experiences of marginalization in health care [23, 24].

Recognizing that Indigenous populations are unique (with longstanding cultural traditions and sovereignty as original inhabitants of territories), there have been efforts to understand how best to serve Indigenous persons in emergency departments [25–27]. There have also been widespread calls for Indigenous communities to deliver or direct their own health services, notably within the *United Nations Declaration of the Rights of Indigenous Peoples* [28]. Yet work in the field of Indigenous emergency care is hindered by a relative lack of comprehensive peer-reviewed statistical analyses, which could impact system level considerations of resource distribution and health services organization.

The objective of this study is to report population rates of emergency care use for FN and non-FN populations, as well as quantitative differences in emergency visit characteristics, between FN and non-FN persons in Alberta, Canada. There are 45 First Nation communities within three treaty areas in Alberta [29], and the province is served by a single health authority [30, 31]. Ability to identify FN patients within emergency care data held by a provincial health authority provides an opportunity to examine system level emergency care statistics across a broad geography (inclusive of rural and urban areas) and varied facility sizes.

## Methods

This project brings together Western and Indigenous research approaches and understandings [32–34]. We recognize that Indigenous knowledge systems encompass quantitative components [35], such as the Blackfoot Winter Count, and that statistical analysis methods can be utilized within research guided by Indigenous principles [36]. Ethics approval is granted by the University of Alberta Health Research Ethics Board Pro 00082440.

This population-based retrospective cohort study examines all emergency care visits in Alberta from April 1, 2012 to March 31, 2017. Data on emergency care visits and urgent care visits are included in the analysis, including where these occur in ambulatory care centres. Three facility types (emergency departments, urgent care centres and ambulatory care centres) provide urgent unscheduled medical care in Alberta, but are not evenly distributed geographically throughout the province. Care that may be provided at an urgent care centre in Edmonton or Calgary could be provided at a small emergency department, or (where one exists) an ambulatory care centre, in a rural or remote area. As such, our provincial analysis of emergency care system use includes these three facility types.

De-identified quantitative administrative data was obtained from the provincial government Ministry ofHealth and the integrated provincial health services provider, Alberta Health Services (AHS) [30, 31], with person-level linkage between datasets complete prior to transfer of the data to researchers. Analytic results were shared regularly with FN partners for collaborative interpretation.

FN-identifying information is based on health care premium payments, which were charged in Alberta until 2009. The federal government paid these fees for registered FN persons, which permitted the identification of FN persons in the Alberta Health Care Insurance Plan (AHCIP) Population Registry. Additionally, an individual (e.g. child) is also flagged as a FN person if they have an AHCIP account for which the main registrant has been identified as a FN person at some point since 1983 [37]. This process of identifying FN persons residing in Alberta is used in government publications of FN health statistics (e.g. [38]) and in academic publications.(e.g. [39]) Alberta Health also provided annual population figures for FN and non-FN populations. “Non-status” FN persons, such as those who lost their federally recognized FN status through colonial policies (see [13] p. 53–55), and status FN persons who came to Alberta from another jurisdiction after 2009, are misidentified as non-FN in the data.

The *National Ambulatory Care Reporting System* (NACRS) collects information on emergency care encounters [40]. Patient age, sex, mode of arrival (e.g. ground ambulance), facility type (described in next paragraph), time and day of emergency care visit, and Canadian Triage Acuity Scale (CTAS) [41] are available within NACRS. CTAS is a triage system used to rate the acuity of the patients’ presenting health condition, in order to prioritize treatment to the most acute urgent cases [41].

Facility types are described by the seven level AHS Peer Groups, which categorize facilities from tertiary hospitals to community ambulatory sites. Tertiary hospitals provide access to specialized services such as organ transplants, high dose chemotherapy and nuclear medicine. Community hospitals provide emergency and inpatient services, and may provide ambulatory care, obstetrics and surgery [42]. Urgent and ambulatory care centres treat urgent conditions that could deteriorate. These centres have no inpatient capacity, and they transport more acute cases that are beyond their capacity to treat [42, 43].

Categorization of patient residence is derived from the AHS “Postal Code” dataset used for geographic analysis. The AHS Rural-Urban Continuum and AHS geographic zones [44] were utilized to analyze distribution of patient residences. The rural-urban continuum stratifies patient residences “by rural/urban status” [45]. The AHS zones are geographic areas organized from North to South. Zones have separate administrative structures, and are used by AHS to organize decision making and delivery of services. Two zones (Edmonton and Calgary) are composed of metropolitan cities and surrounding communities [46, 47], while three are larger geographic areas containing one to two regional centres each (North, Central and South Zones) [48–50].

AHS Distance Tables provide information on the distance in minutes of drive time between patient postal codes and emergency departments, based upon geographic analysis of Alberta’s road network [51]. Patient comorbidities are assessed using the Charlson Comorbidity Index, plus hypertension. The Charlson Index provides the relative “weight” of various combinations of comorbidities based on adjusted mortality and resource use [52]. The AHS Analytics team generated Charlson scores for this project for each emergency visit based on ICD-10 coding of diagnoses using inpatient and NACRS data from a 2 year retrospective time period.

3 M’s Episode Disease Category (EDC) [53] system is utilized to group emergency visit diagnoses. Related EDC categories have been grouped by a physician team member (CB) and the 45 resulting groupings were validated by a second physician team member (BRH; see Additional file 1: Appendix 1).

### Analysis

Age and sex standardized emergency care visit rates per person are derived using 2012/2013 figures as the reference population. Upper and lower 95% confidence interval limits are provided based on standard rates adjusted for recurrent events [54]. Two significant digits are given for population rates, to aid readers who wish to convert these rates to larger denominators (e.g. per 100 persons). Numerical summaries (i.e., means, medians, standard deviations [SDs], IQRs represented as [25th percentile, 75th percentile]) and counts (with percentages) describe emergency care patient demographics and visit data. Analyses at the patient-level use the first emergency visit for patients with multiple visits. All analyses are provided for the FN and non-FN populations separately. Linear mixed-effects models and generalized linear mixed-effects models assess differences between groups, adjusted for repeated emergency visits per patient. Statistical analyses were conducted in R [55] and SAS software [56].

## Results

Alberta Health estimates Alberta’s non-FN population as 4,033,045 in 2017, having increased from 3,714,268 in 2013. Alberta Health estimates the FN population of Alberta as 162,868 in 2017, an increase from 160,123 in 2013. Table 1 shows emergency care visit population rates provincially and across regions of the province (AHS zones). Provincially the age and sex standardized emergency care visit rate is 2.86 times higher for FN.

**Table 1:** Age and sex standardized emergency care visit rates for 2016/2017 (by zone)

Area	FN and Non-FN	ED Visits	2017 Population	Crude Rate per person	Stand. Rate per person	Lower Confidence Interval^a^	Upper Confidence Interval
Province	Non-FN	2,055,318	4,033,045	0.51	0.51	0.51	0.51
	FN	219,490	162,868	1.35	1.45	1.43	1.47
North	Non-FN	412,775	424,030	0.97	0.98	0.98	0.99
	FN	99,553	53,471	1.86	2.01	1.98	2.05
Edmonton	Non-FN	502,291	1,302,619	0.39	0.38	0.38	0.39
	FN	36,385	43,408	0.84	0.89	0.86	0.91
Central	Non-FN	309,449	451,303	0.69	0.67	0.67	0.68
	FN	31,174	20,979	1.49	1.63	1.57	1.68
Calgary	Non-FN	610,684	1,569,841	0.39	0.39	0.39	0.39
	FN	29,250	29,820	0.98	1.04	1.01	1.07
South	Non-FN	167,646	285,252	0.59	0.58	0.57	0.58
	FN	22,016	15,192	1.45	1.57	1.50	1.63

*FN* First Nations, *non-FN* non-First Nations, *ED* Emergency Department

^a^Lower and upper 95% confidence interval limits for standard rate based on adjustment for recurrent events

**Fig. 1:**
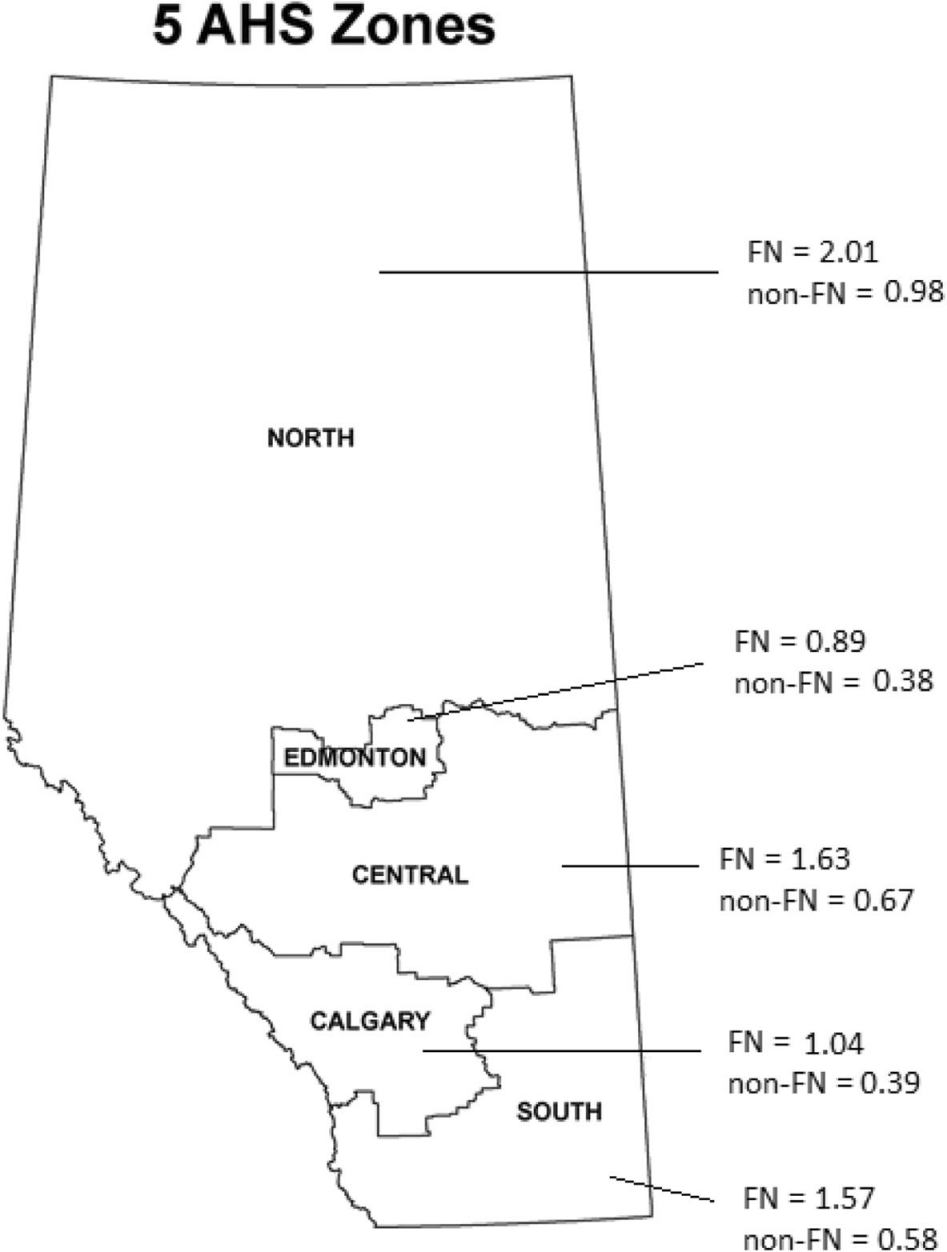
Age and sex standardized emergency care visit rates for 2016/2017 per 1 population, by zone (authors’ own work)

Figure 1 shows 2016/2017 emergency visit rates in the five AHS zones. Rates are higher for FN populations than non-FN populations in all zones. Rates for both population groups are notably higher in the South, Central and North zones compared to Edmonton and Calgary zones. “Rural-urban continuum” categories (e.g. remote, metro) were also examined and FN populations were found to use emergency care more than non-FN populations within all categories.

**Fig. 2:**
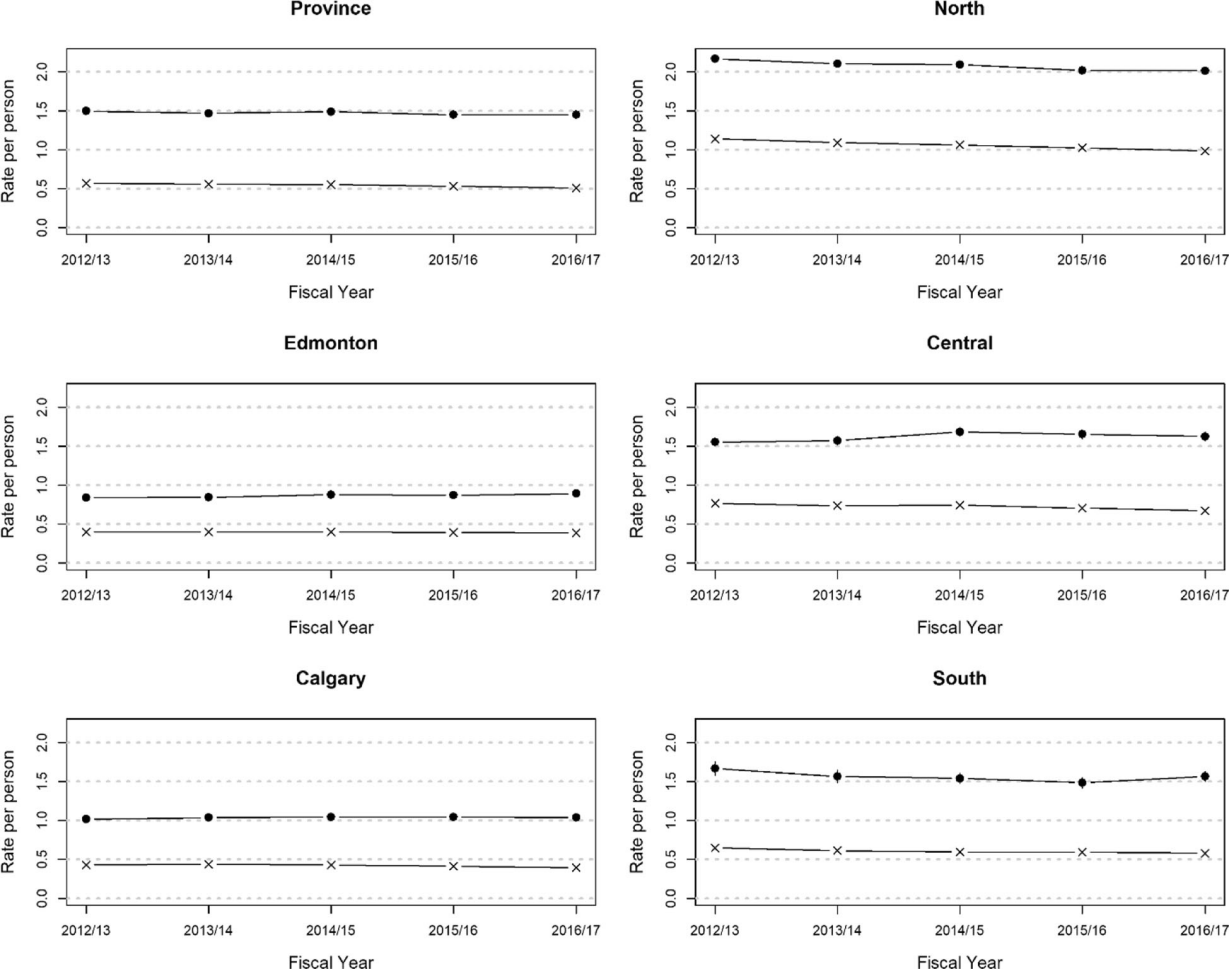
Age and sex standardized emergency care visit rates by zone and fiscal year

Figure 2 shows standardized emergency visit rates provincially and by zone over 5 years for both population groups. The large differences in emergency visit population rates do not narrow over the 5year study period.

Table 2 describes emergency visit categories. Our data set contains 3,024,491 unique patients, of whom 145,508 (4.8%) are FN. These FN patients made 1,099,424 (9.4%) of the 11,686,287 emergency care visits during the study period. Eighty-nine percent of all FN persons used emergency care at least once during our 5 year period (145,508 unique patients divided by 162,868 (2017 FN population)), compared to 71 % of all non-FN persons (2,878,983 unique non-FN patients divided by 4,033,045 (2017 non-FN population)). Individual FN patients who use emergency care utilize it more (median 4 visits per person, [IQR 2, 9]) compared to non-FN patients who utilized emergency care (median 2, [IQR 1, 4]).

**Table 2:** Emergency Care Visit Characteristics^a^

Variable	FN *n* = 1,099,424		Non-FN *n* = 10,586,863	
**Number of unique patients**	145,508	4.8	2,878,983	95.2
**ED visits per patient (over study period)**				
Median [IQR]	4	[2, 9]	2	[1, 4]
**Sex, n %**^b^				
Female	596,248	54.2	5,384,233	50.9
Male	503,173	45.8	5,202,606	**49.1**
**Age**^c^				
Mean (SD)	31.4	(20.2)	38.4	(24.3)
Median [IQR]	30	[17.0 46.0]	36	[20.0 57.0]
Missing, n %	6	0.0	35	0.0
**Visit Day, n %**				
Monday-Friday	791,898	72.0	7,629,544	72.1
Saturday-Sunday	307,526	28.0	2,957,319	27.9
**Visit Time, n %**				
8:01–16:00	488,736	44.5	5,320,816	50.3
16:01–00:00	479,531	43.6	4,029,075	38.1
00:01–08:00	113,157	11.9	1,236,972	11.7
**Ambulance arrival, n (%)**				
Arrive by Ambulance – Air	1325	0.1	5738	0.1
Arrive by Ambulance – Ground	168,553	15.3	1,056,100	10.0
Arrive by Ambulance – Combined	3586	0.3	4446	0.0
Not Admitted by Ambulance	925,960	84.2	9,520,579	89.9
**Triage level, n (%)**				
CTAS 1 – Resuscitation	5284	0.5	45,625	0.4
CTAS 2- Emergent	86,155	7.8	1,208,333	11.4
CTAS 3 – Urgent	306,481	27.9	3,649,685	34.5
CTAS 4 – Less Urgent	446,945	40.7	4,031,673	38.1
CTAS 5 – Non-Urgent	201,399	18.3	1304,155	12.3
No CTAS Assigned	53,160	4.8	347,392	3.3
**Charlson Comorbidity Index Score n (%)**				
0	766,450	69.7	7,914,320	74.8
1	190,737	17.3	1,425,386	13.5
2	61,119	5.6	524,217	5.0
3	29,075	2.6	270,587	2.6
4	16,946	1.5	152,535	1.4
5	9363	0.9	84,064	0.8
6	8310	0.8	55,799	0.5
7 or higher	17,424	1.6	159,955	1.5
**Episode Disease Category Groups (top 10 by FN count) n %**				
Infection	203,045	18.5	1,652,620	15.6
Trauma and Injury	186,562	17.0	2,053,672	19.4
Unspecific Signs, Symptoms, and Findings	125,594	11.4	1,113,140	10.5
Gastrointestinal Conditions	97,940	8.9	1,111,655	10.5
Respiratory Conditions	56,044	5.1	491,546	4.6
Cancer	49,551	4.5	454,915	4.3
Substance Misuse/Addictions	43,895	4.0	97,682	0.9
Breast, Obstetrics and Gynecology	38,857	3.5	299,619	2.8
Musculoskeletal and Arthritis Conditions	34,030	3.1	399,884	3.8
Mental Health	31,938	2.9	245,230	2.3
**Disposition n %**				
Discharged	934,194	85.0	9,167,120	86.6
Admitted	70,295	6.4	833,609	7.9
Transferred	20,249	1.8	191,180	1.8
Left Without Being Seen	48,690	4.4	290,468	2.7
Left Against Medical Advice	25,540	2.3	97,877	0.9
Deceased	456	0.0	6609	0.1

*FN* First Nations, *non-FN* non-First Nations, *ED* Emergency Department, *IQR* interquartile range, *SD* standard deviation, *CTAS* Canadian Triage Acuity Score

^a^All difference between FN and non-FN are significant at the p = < 0.001 level with one exception. Visit day is not significantly different (*p* = 0.40)

^b^< 30 additional visits by FN and non-FN patients had the sex of the patient recorded as “Other.” The low number may be due to inconsistent use of this category rather than a reflection of the population

^c^Patient age in years as calculated at admission. Ages> 110 were recoded as missing

Table 3 describes the geographic and facility type distribution of ED visits. One hundred and eleven facilities had emergency care visits during our study period. FN emergency visit numbers to individual facilities varied (median 3279, [IQR 960, 14,338]). Thirty-three (30% of 111) facilities saw 80% of all FN visits provincially. In four facilities, > 50% of visits were made by FN patients. The highest percentage of FN visits, as a proportion of visits to a facility, was 83% (43,739 of 52,844 total visits), while the lowest was 0.4% (70 FN visits of 18,358 total visits).

**Table 3:** Visit Geography^a^

Variable	FN *n* = 1,099,424		Non-FN *n* = 10,586,863	
**Drive time from patient postal code to nearest Emergency Department (minutes)**				
Mean (SD)	17.8	(25.2)	9.9	(9.6)
Median [IQR]	9.0	[3, 23]	7.0	[3, 13]
Missing	44,378		425,628	
**Drive distance from patient postal code to Emergency Department (km)**				
Mean (SD)	19.0	(32.1)	7.6	(11.1)
Median [IQR]	6.0	[1, 24]	4.0	[2, 8]
Missing	44,378		425,628	
**Urban / Rural Continuum (Patient Address) n %**				
Metro (population > 500,000)	178,715	16.3	3,673,570	34.7
Moderate Metro Influence (immediately surrounding Metro Centres)	49,293	4.5	1,338,763	12.6
Urban (Population > 25,000 but less than > 500,000)	66,115	6.0	1,064,243	10.1
Moderate Urban Influence (surrounding urban centres)	5717	0.5	198,378	1.9
Rural Centre Area (> 10,000 to less than < 25,000)	150,974	13.7	538,840	5.1
Rural (population less than 10,000 and up to 200 km from a Metro or Urban Centre)	377,692	34.4	2,888,163	27.3
Rural Remote (> 200 km from a Metro or Urban Centre)	234,735	21.4	471,716	4.5
Missing	36,183	3.3	413,190	3.9
**Patient Address Area - AHS Zone n %**				
North	509,990	46.4	2,195,754	20.7
Edmonton	174,515	15.9	2,455,812	23.2
Central	156,924	14.3	1,639,139	15.5
Calgary	143,377	13	3,106,590	29.3
South	108,277	9.8	862,415	8.1
Missing	6341	0.6	327,153	3.1
**Facility Type n %**				
Tertiary Referral	124,221	11.3	1,598,194	15.1
Regional Referral	183,055	16.7	3,152,477	29.8
Community Hospital providing 5 services with < 5000 Inpatient Discharges per year	357,147	32.5	2,132,601	20.1
Community Hospital providing 3 to 4 services – or providing Emergency and Inpatient with > 600 emergency inpatient discharges per year.	226,897	20.6	1,437,449	13.6
Community Hospital providing Emergency and Inpatient Services with < 600 inpatient discharges per year.	131,703	12.0	804,591	7.6
Community Ambulatory Moderate > = 20,000 visits per year	21,337	1.9	356,555	3.4
Community Ambulatory < 20,000 visits per year	19,102	1.7	158,719	1.5
Urgent Care Centre	35,962	3.3	946,277	8.9

*FN* First Nations, *non-FN* non-First Nations, *ED* Emergency Department, *SD* standard deviation, *IQR* interquartile range, *AHS* Alberta Health Services

^a^All difference between FN and non-FN are significant at the p = < 0.001 level

FN women (female sex) use the emergency care system proportionately more than their non-FN counterparts, while FN men use the emergency care system comparatively less than both FN women and non-FN men. Patient age across FN visits differs by a median of 6 years from patient age across non-FN visits (FN median 30, [IQR 17, 46]; non-FN median 36, [IQR 20, 57]). FN visits end more often in leaving without being seen or against medical advice (6.7% of FN visits vs. 3.6% of non-FN visits) and are more often in the evening (43.6% vs. 38.1%). See Additional file 1: Appendix 2 for a table of emergency visits by day of the week and by ED shift. FN visits arrive more often by ground ambulance (15.3% vs. 10%) relative to non-FN visits and are more commonly triaged as less acute (59% CTAS 4 (less urgent) and 5 (non-urgent), compared to 50.4% non-FN). The top 10 groups of episode disease categories by FN count comprise 78.9% of all FN visits during the study period. Among these ten categories, trauma and injury, gastrointestinal conditions, and musculoskeletal and arthritis conditions, make up a lower percent of FN than non-FN visits. FN emergency visits have higher comorbidity scores than non-FN emergency visits. Additional file 1: Appendix 3 presents the incidence of specific comorbidities across emergency visits. Additional file 1: Appendix 4 presents characteristics of unique patients (as opposed to emergency visits), based on a patient’s first visit. Patient level statistics show higher emergency care use by patients who are older, live closer to emergency facilities, and have higher comorbidity scores for both FN and non-FN patients. FN women do not make up as large a proportion of unique FN patients (50.7%), as they do of FN emergency visits (54.2%). This finding demonstrates that FN women who use emergency care had more visits per person than FN men.

## Discussion

### Explanation of findings

FN persons make up 4% of the Alberta population but 9.4% of all emergency visits. 89% of the FN population visited emergency care at least once during our study period compared to 71% of the non-FN population. This proportion shows that FN patients are an important patient group for emergency care.

Five FN Elder knowledge holders and five FN Health Directors co-interpreted data with researchers. They contextualized higher emergency care visit rates in terms of a lack of access to primary care. Our study did not examine primary care access in relation to ED use, and further research would be needed to examine links between primary care access and emergency care quantitatively. However, wider literature suggests a link between access to high quality primary care and emergency care use [57–60]. Studies also suggest that FN access to primary care is limited [61–66] and so a case may be made for the need for such further research. Indeed, a recent government inquiry into Indigenous health care in the neighboring province of British Columbia directly compares use of emergency care to primary care access, and found that Indigenous people who were not attached to primary care “were more likely to visit the ED and be hospitalized.” [60] Similarly, innovative analysis by Lavoie and colleagues shows that levels of primary health care in FN communities are associated with reductions in avoidable hospitalizations and premature mortality [65]. Further research reproducing their methods could be conducted to determine if emergency care use is similarly impacted by levels of primary care access within communities.

In our study, we also found that a large proportion of FN visits are triaged as lower acuity (CTAS 4 and 5), and this finding could be related to use of emergency care for primary care concerns. However, it should be noted that Zook and colleagues [67] have reported under-triage of American Indian patients based on race, and further research would be needed to explore triage of First Nations patients in Canada.

Our results also show that FN women present to emergency care more than non-FN women. Turpel-Lafond has similarly found that Indigenous women utilize emergency departments more than Indigenous men in British Columbia [60]. In meetings for our project, Elders hypothesized that FN men may present to emergency care less than FN women because of a greater reluctance to seek care among FN men. By contrast, Turpel-Lafond concluded that Indigenous women face unique forms of discrimination, and that their greater emergency care use is driven in part by greater avoidance of preventive and primary health care [60]. Turpel-Lafond also found that Indigenous women face a health care gap, compared to non-Indigenous women, in terms of such services as midwifery, obstetrician deliveries and antenatal visits [60]. Further research on FN women’s emergency care in Alberta is warranted.

FN team members contextualized presentations to emergency care in evenings and arrival by ambulance in terms of issues accessing transport to and from emergency facilities. A 2005 study by Wallace and colleagues similarly found that use of emergency care may be associated with lack of access to non-urgent medical transportation [68].

FN partners contextualized leaving without completing treatment in terms of experiences of discrimination within the health care system (see also [23, 69]), needing to leave when transport is available and leaving care to fulfill family responsibilities. Issues related to discrimination, transport and family responsibilities could be explored through survey or qualitative research with FN users of emergency care.

Findings of higher rates of FN patients leaving without being seen and leaving against medical advice are similar to earlier US findings for American Indian populations [22, 70], for FN Chronic Obstructive Pulmonary Disorder patients in Alberta emergency departments [21], and for FN abdominal pain patients in a Saskatchewan emergency department [20]. Patients who leave emergency care without completing treatment may be at risk of returning to emergency facilities [71] and admissions to hospital [71, 72]. To our knowledge, no studies have examined outcomes for FN patients who leave without completing treatment. This is an important area for future research.

High use of emergency care does not necessarily mean FN patients are using emergency care unnecessarily or inappropriately [73]. There is no agreed upon definition of what constitutes inappropriate or non-urgent emergency care use [74, 75]. Patients choose emergency care because it is the right care option from their perspective [76].

### Future directions

As noted above, links between FN emergency care use and access to other components of the health care system (especially primary care), emergency care of FN women, factors impacting emergency care use such as anti-Indigenous discrimination and access to non-emergency transport, and emergency care outcomes for FN patients (including those who leave without completing treatment) are important areas for future research. To address differences in patient demographics, future research should examine outcomes reported here stratified by sex and geography, and qualitative research should examine different groups’ understandings of quantitative findings (e.g. FN men and women, rural and urban FN persons). In addition, as many factors interact to determine FN patients’ emergency care visit characteristics and outcomes, further quantitative research should attempt to separate the impact of FN identity from factors such as diagnosis, distance traveled to care and size of facility presented to. Stratifying analysis by any of these variables would produce distinct descriptive statistical results, which may be of value for specific projects or more granular understanding of FN emergency care. The provincial analysis reported here may aid in making decisions about which variables to stratify by in such future analysis.

### Limitations

Our data counts most non-status FN persons, status FN persons who came to Alberta after 2009, and potentially some status FN persons not identified in the data set for other unknown reasons, as non-FN persons. As such, differences between FN and non-FN statistics may be under-reported. Future research could overcome this limitation if access to the Federal “Indian Register” could be obtained [77]. However, our use of the provincial data identifiers that are relied upon by Alberta Health in its reporting on FN health outcomes provides comparability to provincial government analyses and so allows our work to better inform health system decision makers.

Métis and Inuit persons are also counted among the non-FN population, concealing any similarities in emergency care statistics among Indigenous populations. This limitation means that we cannot say how FN emergency care statistics compare to other Indigenous populations’ emergency care statistics. We chose to work with FN for this project, recognizing their distinct history, governance structures and governing relationships with Federal and Provincial jurisdictions compared to other Indigenous peoples in Alberta. If future research involved Métis governing bodies and appropriate Inuit partners, and data identifiers were available, this limitation could be overcome.

Geographic data represent only a point in time. Patients may move more frequently than this data is updated. When accessing emergency care, patients will not always be travelling from their residence or to the facility closest to their residence. If FN and non-FN patients differ systematically in frequency of moving residence or number of emergency care visits where the patient travels to the nearest emergency department from their residence, this would impact our results. We are not aware of quantitative data that suggests such systematic differences. Limitations on geographic data derive from provincial health administrative data relied on for this study. Future research with a smaller sample and utilizing primary data collection could overcome these limitations by verifying up to date residence information and by deriving travel distances between hospital addresses for specific emergency care visits and patient residences.

## Conclusions

FN persons’ rely on the emergency care system more than non-FN persons in Alberta. High rates of FN visits ending without completing treatment are concerning as this outcome may indicate dissatisfaction with care, and has been found in some studies to expose patients to increased odds of hospital admission. Future research could productively examine emergency care outcomes for FN patients (including for those who leave emergency care without completing treatment), explore links between FN emergency care use and access to other health services (especially primary care), stratify quantitative results reported here by variables such as geography (e.g. rural vs urban) or facility type, and examine FN women’s emergency care. Qualitative research to obtain FN perspectives on quantitative findings will also be invaluable for better understanding emergency care of FN populations.

## Supplementary Information

**Additional file 1: Appendices 1-4. 1.** EDC Groupings used in Analysis. **Appendix 2.** Day of Week by Shift (Night, Day, Evening). **Appendix 3.** Emergency visit comorbidities. **Appendix 4.** Unique Patient Variables (based on 1st emergency visit).

## Data Availability

The original data sources for this study are owned by Alberta Health Services and Alberta Health. Readers may contact the corresponding author for more information.

## Abbreviations

FN: First Nations
CTAS: Canadian Triage Acuity Score
AHS: Alberta Health Services
AHCIP: Alberta Health Care Insurance Plan
NACRS: National Ambulatory Care Reporting System
EDC: Episodic Disease Category
