# Supplementary material for: First Nations emergency care in Alberta: descriptive results of a retrospective cohort study
Source: BMC Health Serv Res. 2021 May 4;21:423. doi: 10.1186/s12913-021-06415-2 (PMC8096356; doi:10.1186/s12913-021-06415-2)
Supplement: Supplementary file 1 — Additional file 1: Appendices 1-4. 1. EDC Groupings used in Analysis. Appendix 2. Day of Week by Shift (Night, Day, Evening). Appendix 3. Emergency visit comorbidities. Appendix 4. Unique Patient Variables (based on 1st emergency visit). [file 12913_2021_6415_MOESM1_ESM.zip › FNED2 Appendices April 13, 2021.docx]

**Appendix 1: EDC Groupings used in Analysis**

| **EDC  Grouping** | **EDC in group** |
| --- | --- |
| (Group) Trauma  and Injury | Abdominal Trauma; Amputation; Burns; Cardiothoracic Trauma; Frostbite; Head and Neck Injuries; Head and Neck Trauma; Kidney or Ureter Trauma; Lower Limb Injuries; Nerve Root and Spinal Plexus Injury; Other Fracture; Other Injuries; Spinal Cord Injury; Spinal Fractures without Spinal Cord Injury |
| (Group) Infection | Cellulitis; Gangrene; Infections; Kidney Infections; Otitis Media; Pneumonia; Recurrent Urinary Tract Infections; Septicemia; Tuberculosis; Upper Respiratory Infections; Urinary Tract Infections;  Venereal Disease; Decubitus Ulcer |
| Unspecific Signs,  Symptoms, and  Findings | No Grouping |
| (Group)  Gastrointestinal  Conditions | Abdominal Pain; Acute Gallbladder Disorder; Acute Pancreatic and Liver Disorders; Appendicitis; Chronic Liver Disorders; Gallbladder Disease; Jaundice; Malnutrition; Nausea, Vomiting, and Diarrhea; Obesity;  Ostomy Status; Stomach and Intestinal Disorders |
| (Group)  Respiratory  Conditions | Acute Respiratory Diseases; Asthma; Chronic Obstructive Pulmonary Disease; Chronic Respiratory Diseases; Pleural Effusion; Cyanosis; Respiratory Anomalies; Respiratory Therapy/Complications |
| (Group) Cancer | Bone Malignancy; Brain and Central Nervous System Malignancies; Breast Malignancy; Cervical Malignancy; Colon Malignancy; Ear, Nose, and Throat Malignancies; Esophageal Malignancy; Genitourinary  Malignancy; Gynecological Malignancies Except Uterine, Cervical, and Ovarian; Hodgkin's Lymphoma; Kaposi's Sarcoma; Kidney Malignancy; Leukemia; Liver Malignancy; Lung Malignancy; Melanoma; Myeloma; Non-Hodgkin's Lymphoma; Other Malignancies; Ovarian Malignancy; Pancreatic Malignancy; Plasma Protein Malignancy; Prostate  Malignancy; Radiation/Chemotherapy; Skin Malignancy; Stomach Malignancy; Uterine Malignancy |
| (Group)  Musculoskeletal  and Arthritis  Conditions | Acute Joint and/or Musculoskeletal Diagnoses; Chronic Joint and Musculoskeletal Diagnoses; Connective Tissue Disease and/or Vasculitis; History of Hip Fracture; Joint Replacement; Orthopedic Devices Complications; Osteoarthritis; Pathological Dislocation; Pathological Fractures and Aseptic Necrosis; Pelvis, Hip, and Femur Deformities;  Rheumatoid Arthritis |
| (Group) Breast,  Obstetrics and  Gynecology | Abortion; Acute Gynecological Diagnoses; Breast Diagnoses; Cesarean Section; Chronic Gynecological Diagnoses; Infertility - Female; Obstetrical Diagnosis; Tubal/Ovarian/Ectopic Pregnancy |
| (Group) Other  Cardiac  Conditions | Cardiovascular Device Status and grafts; Chest Pain; Chronic Cardiovascular Diagnoses; Coronary Atherosclerosis. History of Coronary Artery Bypass Graft; History of Percutaneous Transluminal Coronary Angioplasty |
| (Group) Spinal  Conditions | Acute Back and Spine Diagnoses; Curvature or Anomaly of the Spine; History of Major Spinal Procedure; Spinal Disc Disease; Spinal Stenosis |

**Appendix 2: Day of Week by Shift (Night, Day, Evening)**

| ***FN, n, % of FN visits*** | | | | | | | | | | | |
| --- | --- | --- | --- | --- | --- | --- | --- | --- | --- | --- | --- |
|  | **00:01-08:00** | | | | **08:01-16:00** | | | **16:01-00:00** | | **Total** | |
| **Sun** | 20475 | | | 1.9% | 66239 | 6.0% | | 67475 | 6.1% | 154189 | 14.0% |
| **Mon** | 18185 | | | 1.7% | 75712 | 6.9% | | 73947 | 6.7% | 167844 | 15.3% |
| **Tue** | 17789 | | | 1.6% | 71657 | 6.5% | | 72225 | 6.6% | 161671 | 14.7% |
| **Wed** | 17852 | | | 1.6% | 70346 | 6.4% | | 69787 | 6.3% | 157985 | 14.4% |
| **Thu** | 17967 | | | 1.6% | 67780 | 6.2% | | 67000 | 6.1% | 152747 | 13.9% |
| **Fri** | 18071 | | | 1.6% | 68964 | 6.3% | | 64616 | 5.9% | 151651 | 13.8% |
| **Sat** | 20818 | | | 1.9% | 68038 | 6.2% | | 64481 | 5.9% | 153337 | 13.9% |
| **Total** | 131157 | | | 11.9% | 488736 | 44.5% | | 479531 | 43.6% | 1099424 | 100.0% |
| ***Non-FN n, % of Non-FN visits*** | | | | | | | | | | | |
|  | | **00:01-08:00** | | | **08:01-16:00** | | **16:01-00:00** | | | **Total** | |
| **Sun** | | | 185553 | 1.8% | 747152 | 7.1% | | 566845 | 5.4% | 1499550 | 14.2% |
| **Mon** | | | 182900 | 1.7% | 836642 | 7.9% | | 607208 | 5.7% | 1626750 | 15.4% |
| **Tue** | | | 175808 | 1.7% | 764190 | 7.2% | | 590804 | 5.6% | 1530802 | 14.5% |
| **Wed** | | | 171938 | 1.6% | 746565 | 7.1% | | 579121 | 5.5% | 1497624 | 14.1% |
| **Thu** | | | 170892 | 1.6% | 737226 | 7.0% | | 570455 | 5.4% | 1478573 | 14.0% |
| **Fri** | | | 173118 | 1.6% | 756200 | 7.1% | | 566477 | 5.4% | 1495795 | 14.1% |
| **Sat** | | | 176763 | 1.7% | 732841 | 6.9% | | 548165 | 5.2% | 1457769 | 13.8% |
| **Total** | | | 1236972 | 11.7% | 5320816 | 50.3% | | 4029075 | 38.1% | 10586863 | 100.0% |

Legend: FN First Nations; non-FN non-First Nations

**Appendix 3: Emergency visit comorbidities**

| **Comorbidities^[[1]](#footnote-1)^** | **FN** | | **Non-FN** | |
| --- | --- | --- | --- | --- |
|  | **n** | **%** | **n** | **%** |
| Chronic Pulmonary Disease | 173,761 | 15.8 | 1,156,560 | 10.9 |
| Hypertension | 115,370 | 10.5 | 1,261,027 | 11.9 |
| Diabetes without Complications | 77,929 | 7.1 | 459,274 | 4.3 |
| Diabetes with complications | 54,399 | 4.9 | 437,239 | 4.1 |
| Mild Liver Disease | 28,391 | 2.6 | 100,008 | 0.9 |
| Peptic Ulcer Disease | 24,526 | 2.2 | 124,812 | 1.2 |
| Congestive Heart Failure | 21,267 | 1.9 | 311,103 | 2.9 |
| Renal Disease | 20,643 | 1.9 | 209,123 | 2.0 |
| Myocardial Infarction | 19,747 | 1.8 | 217,676 | 2.1 |
| Cancer | 19,383 | 1.8 | 355,399 | 3.4 |
| Rheumatic Disease | 17,879 | 1.6 | 122,109 | 1.2 |
| Cerebrovascular Disease | 17,593 | 1.6 | 249,238 | 2.4 |
| Moderate or Severe Liver Disease | 12,576 | 1.1 | 44,834 | 0.4 |
| Peripheral Vascular Disease | 11,309 | 1.0 | 148,999 | 1.4 |
| HIV | 6,932 | 0.6 | 8,581 | 0.1 |
| Paraplegia | 6,868 | 0.6 | 56,173 | 0.5 |
| Metastatic Carcinoma | 6,305 | 0.6 | 120,526 | 1.1 |
| Dementia | 4,508 | 0.4 | 122,856 | 1.2 |

Legend: FN First Nations; non-FN non-First Nations

**Appendix 4**: **Unique Patient Variables (based on 1^st^ emergency visit)**

| **Variable** | | FN  n=145,508 | | Non-FN  n=2,878,983 | |
| --- | --- | --- | --- | --- | --- |
| **Sex, n %** | |  |  |  |  |
|  | Female | 73,822 | 50.7 | 1,405,224 | 48.8 |
|  | Male | 71,686 | 49.3 | 1,473,754 | 51.2 |
| **Age (years)** | |  |  |  |  |
|  | Mean (SD) | 26.3 | (19.8) | 35.7 | (23.4) |
|  | Median [IQR] | 23.0 | [10, 41] | 33.0 | [18, 53] |
|  | Missing n, % | 2 | 0.0 | 25 | 0.0 |
| **Drive time from patient postal code to nearest Emergency Department (minutes)** | |  |  |  |  |
|  | Mean, (SD) | 18.4 | (26.6) | 10.7 | (8.9) |
|  | Median [IQR] | 10 | [4, 23] | 9.0 | [4, 14] |
|  | Missing | 5,706 |  | 210,005 |  |
| **Drive distance from patient postal code to Emergency Department (km)** | |  |  |  |  |
|  | Mean, (SD) | 19.2 | (33.9) | 7.7 | (10.3) |
|  | Median [IQR] | 6 | [2, 22] | 5 | [2, 9] |
|  | Missing | 5,706 |  | 210,005 |  |
| **Urban influence area n,%** | |  |  |  |  |
|  | Metro | 34,093 | 23.4 | 1,255,547 | 43.6 |
|  | Moderate Metro Influence | 10,054 | 6.9 | 383,963 | 13.3 |
|  | Urban | 10,754 | 7.4 | 284,525 | 9.9 |
|  | Moderate Urban Influence | 1,184 | 0.8 | 58,761 | 2.0 |
|  | Rural Centre Area | 18,607 | 12.8 | 116,642 | 4.1 |
|  | Rural | 43,157 | 29.7 | 504,513 | 17.5 |
|  | Rural Remote | 23,567 | 16.2 | 67,449 | 2.3 |
|  | Missing | 4,092 | 2.8 | 207,583 | 7.2 |
| **Patient Address Area - AHS Zone n %** | |  |  |  |  |
|  |  |  |  |  |  |
|  | North | 53,938 | 37.1 | 376,976 | 13.1 |
|  | Edmonton | 31,039 | 21.3 | 780,460 | 27.1 |
|  | Central | 20,322 | 14 | 343,454 | 11.9 |
|  | Calgary | 23,954 | 16.5 | 977,389 | 33.9 |
|  | South | 14,020 | 9.6 | 211,114 | 7.3 |
|  | Missing | 4,092 | 2.8 | 207,583 | 7.2 |
| **Charlson Comorbidity Index Score, n %** | |  |  |  |  |
|  | 0 | 126,383 | 86.9 | 2,516,262 | 87.4 |
|  | 1 | 13,497 | 9.3 | 234,603 | 8.1 |
|  | 2 | 3,019 | 2.1 | 68,830 | 2.4 |
|  | 3 | 1,117 | 0.8 | 26,524 | 0.9 |
|  | 4 | 571 | 0.4 | 11,076 | 0.4 |
|  | 5 | 263 | 0.2 | 4,730 | 0.2 |
|  | 6 | 232 | 0.2 | 4,057 | 0.1 |
|  | 7 or higher | 426 | 0.3 | 12,901 | 0.4 |
| **Co-morbidities, n %** | |  |  |  |  |
|  | Chronic Pulmonary Disease | 9,107 | 6.3 | 125,336 | 4.4 |
|  | Diabetes without Complications | 5,230 | 3.6 | 85,303 | 3.0 |
|  | Hypertension | 5,305 | 3.6 | 146,341 | 5.1 |
|  | Diabetes with complications | 2,186 | 1.5 | 43,172 | 1.5 |
|  | Cancer | 883 | 0.6 | 42,071 | 1.5 |
|  | Peptic Ulcer Disease | 911 | 0.6 | 10,694 | 0.4 |
|  | Cerebrovascular Disease | 876 | 0.6 | 32,174 | 1.1 |
|  | Mild Liver Disease | 844 | 0.6 | 9,325 | 0.3 |
|  | Myocardial Infarction | 802 | 0.6 | 21,093 | 0.7 |
|  | Rheumatic Disease | 803 | 0.6 | 13,456 | 0.5 |
|  | Congestive Heart Failure | 758 | 0.5 | 25,738 | 0.9 |
|  | Renal Disease | 732 | 0.5 | 17,329 | 0.6 |
|  | Peripheral Vascular Disease | 343 | 0.2 | 12,502 | 0.4 |
|  | Paraplegia | 344 | 0.2 | 5,558 | 0.2 |
|  | Moderate or Severe Liver Disease | 293 | 0.2 | 2,762 | 0.1 |
|  | Metastatic Carcinoma | 235 | 0.2 | 12,292 | 0.5  0.4 |
|  | Dementia | 203 | 0.1 | 13,916 | 0.5 |
|  | HIV | 169 | 0.1 | 947 | 0 |

Legend: FN First Nations; non-FN non-First Nations; SD standard deviation; IQR interquartile range; AHS Alberta Health Services

1. These statistics are not age standardized. [↑](#footnote-ref-1)
